# Supplementary figures and images for: Deep learning algorithm in detecting intracranial hemorrhages on emergency computed tomographies
Source: PLoS One. 2021 Nov 29;16(11):e0260560. doi: 10.1371/journal.pone.0260560 (PMC8629230; doi:10.1371/journal.pone.0260560)

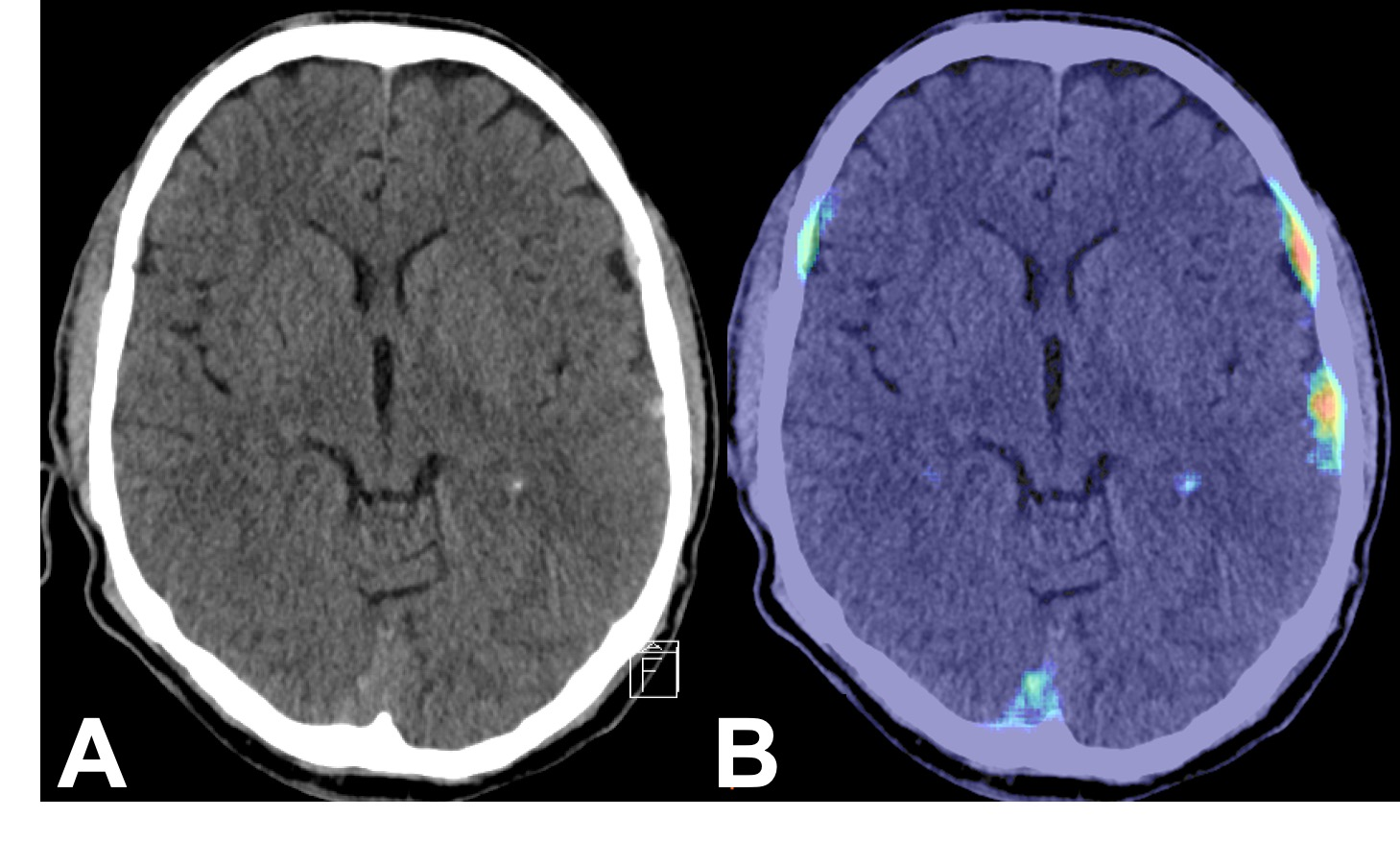

Supplement: S1 Fig — A: Axial HCT showing a thin acute SDH and small contusion of the right temporal hemisphere. B: Corresponding color-coded map identifying the ICHs correctly as main findings. These ICHs were missed by the RR (RR, radiology report; HCT, head computed tomography; SDH, subdural hematoma; ICH, intracranial hemorrhage). (TIF) [file pone.0260560.s002.tif]

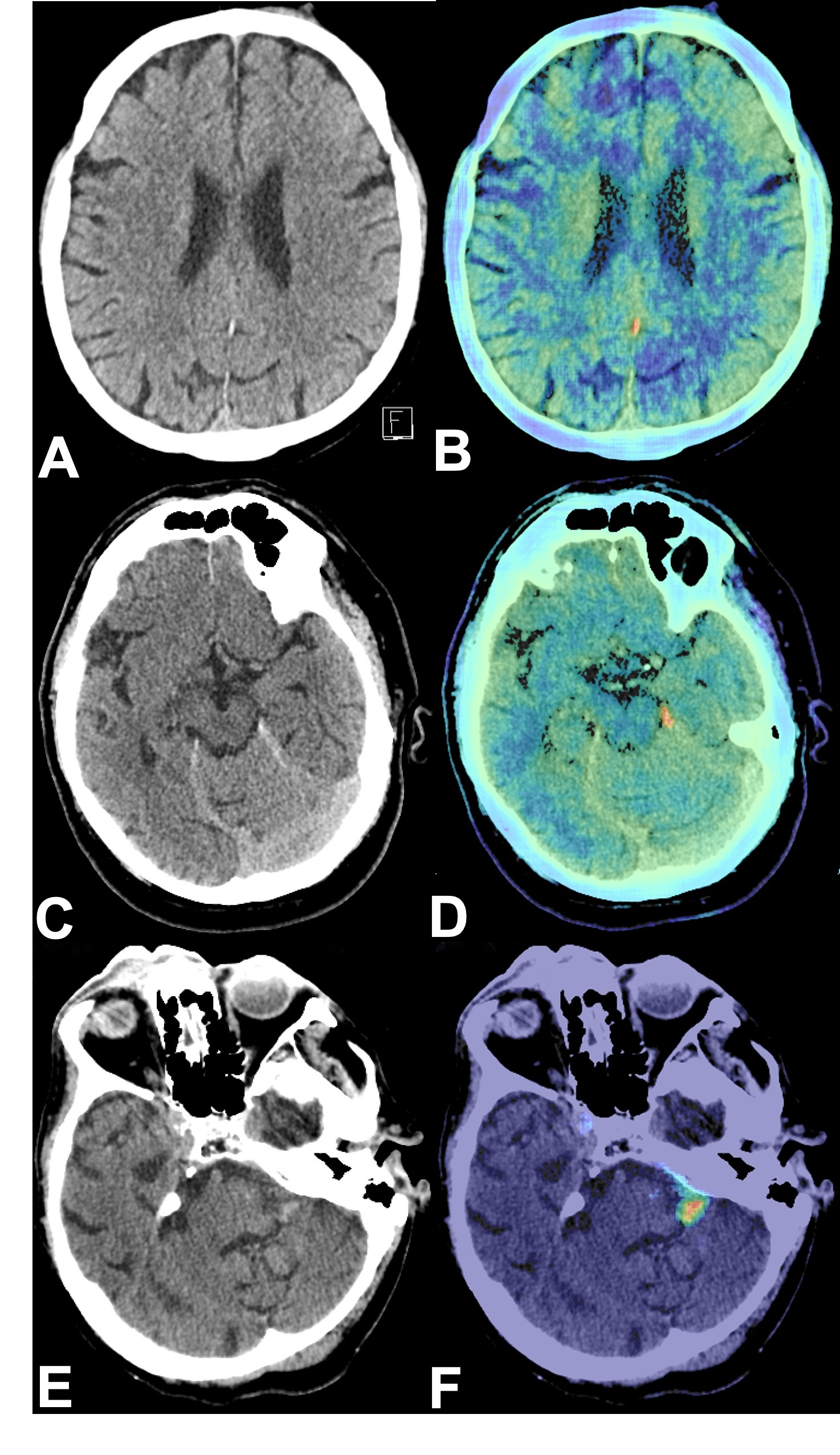

Supplement: S2 Fig — A, C, E: Axial HCT without ICH, typical false positive results. Color-coded maps flagging a calcified spot of the falx (B), part of the tentorium (D), and part of the infratentorial plexus at the left lateral aperture (F) (HCT, head computed tomography; ICH, intracranial hemorrhage). (TIF) [file pone.0260560.s003.tif]

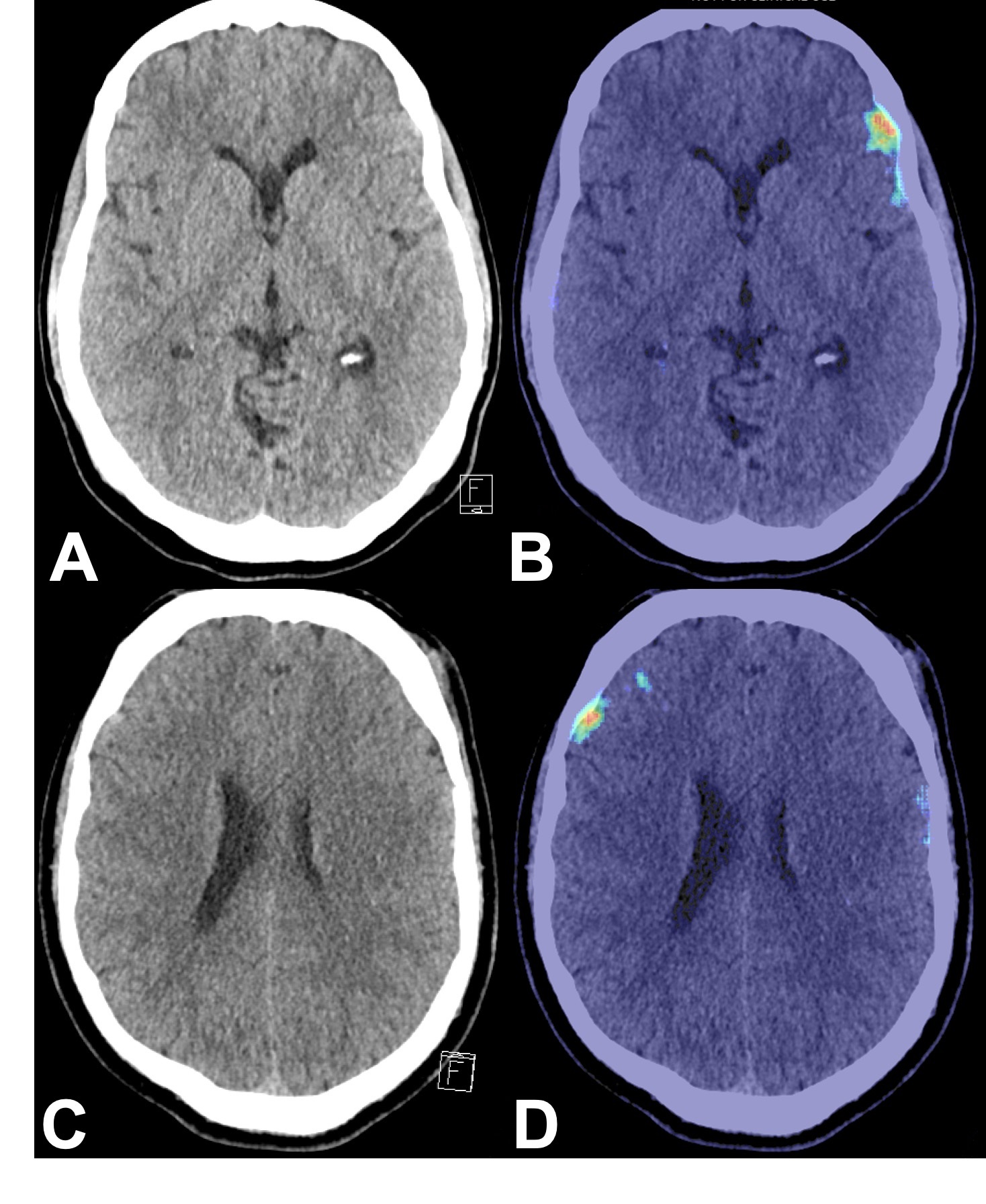

Supplement: S3 Fig — A, C: Axial HCT without ICH. B and D: Corresponding color-coded maps with false positive findings underneath the frontal skull due to beam-hardening artifacts (HCT, head computed tomography; ICH, intracranial hemorrhage). (TIF) [file pone.0260560.s004.tif]

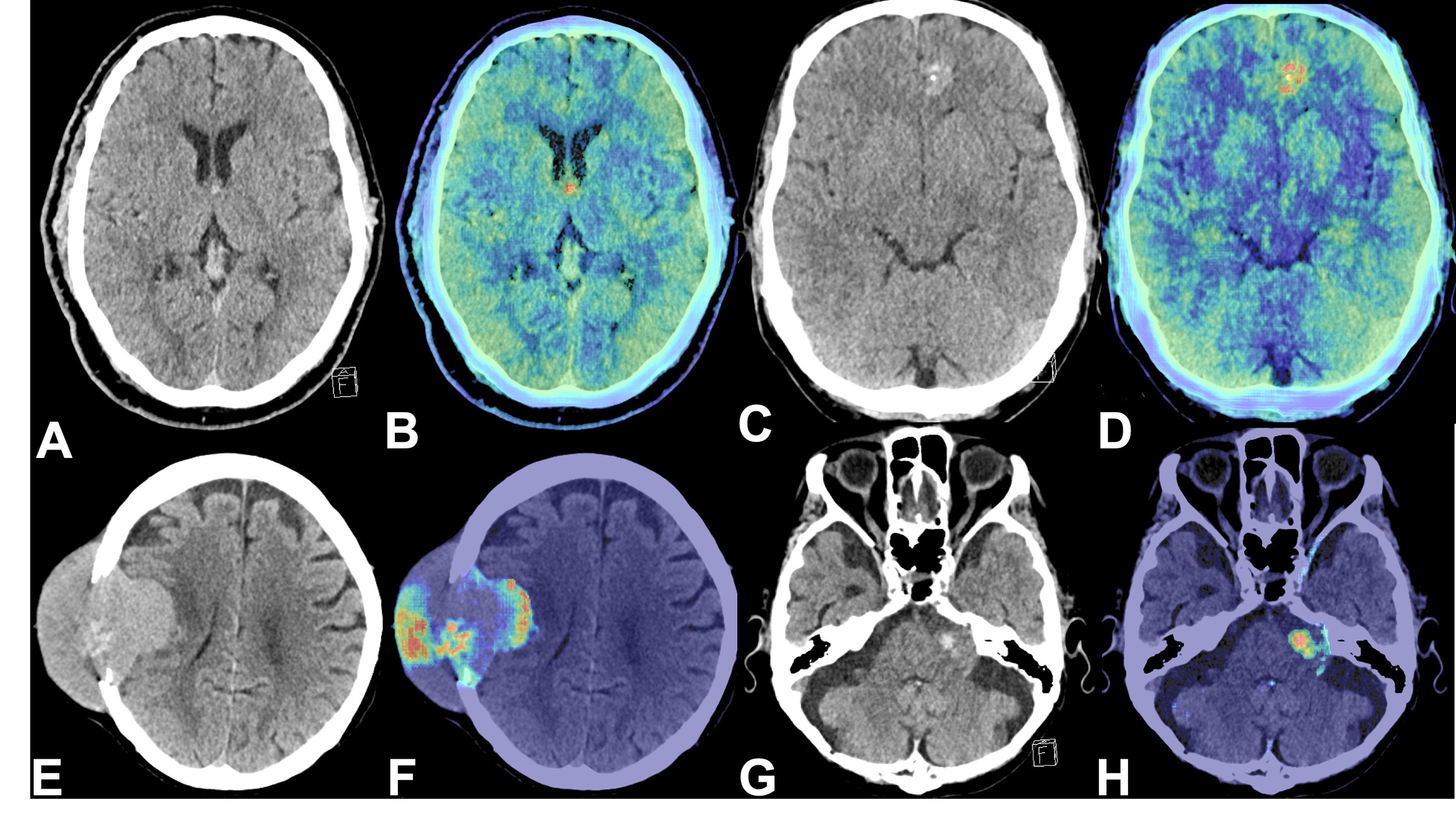

Supplement: S4 Fig — A, C, E, F: Axial HCT showing hyperdense/partially calcified tumors. A: colloid cyst of the third ventricle, E: intra-/extracranial metastasis of the right frontoparietal hemisphere, G: vestibular schwannoma of the left auditory canal with extra-/intra-canicular growth. B, C, G: Color-coded maps flagging the tumors (HCT, head computed tomography). (TIF) [file pone.0260560.s005.tif]

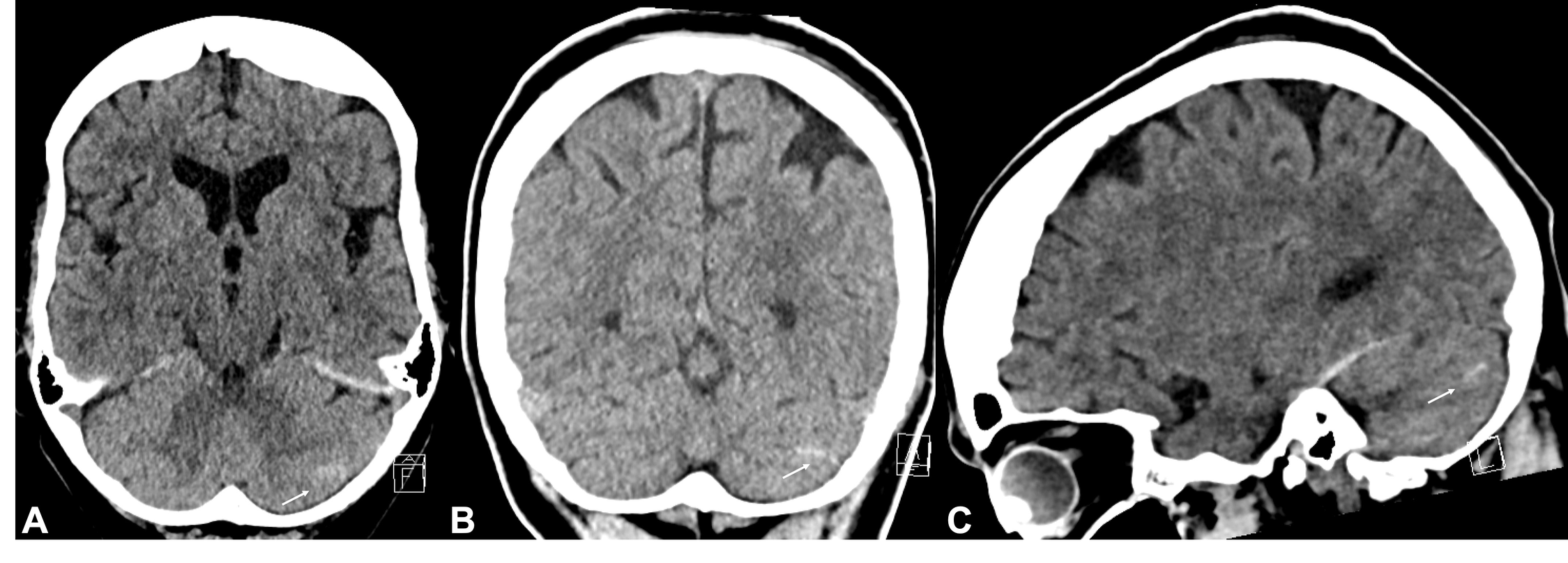

Supplement: S5 Fig — Axial (A), coronal (B), and sagittal (C) reconstructions showing a thin SAH of the left cerebellar hemisphere (white arrows) which was not flagged by the algorithm (SAH, subarachnoid hemorrhage). (TIF) [file pone.0260560.s006.tif]
