# Supplementary material for: Deep learning algorithm in detecting intracranial hemorrhages on emergency computed tomographies
Source: PLoS One. 2021 Nov 29;16(11):e0260560. doi: 10.1371/journal.pone.0260560 (PMC8629230; doi:10.1371/journal.pone.0260560)
Supplement: S1 Table — Characteristics of the whole case mix divided into cases with and without intracranial hemorrhage according to the neuroradiologist. IQR, interquartile range; ICH, intracranial hemorrhage; CT, computed tomography. (DOCX) [file pone.0260560.s007.docx]

**S1 Table. Characteristics of included cases and scanning environment.**

| **Variable** | **Total** | **ICH** | **No ICH** |
| --- | --- | --- | --- |
| n | 4946 | 267 | 4679 |
| Median age, years (IQR) | 72 (56-83) | 71 (58-82) | 72 (56-83) |
| Gender, n (%) |  |  |  |
| Male | 2596 (52.5%) | 161 (60.3 %) | 2435 (52.0 %) |
| Female | 2347 (47.5%) | 106 (39.7 %) | 2241 (47.9 %) |
| Missing | 3 (0.0%) |  | 3 (0.0%) |
| Location of study site |  |  |  |
| In-House | 2736 (55.3 %) | 173 (64.8 %) | 2563 (54.8 %) |
| Teleradiology | 2210 (44.7 %) | 94 (35.2%) | 2116 (45.2 %) |
| CT technique |  |  |  |
| Incremental | 902 (18.2%) | 38 (14.2%) | 864 (18.5%) |
| Spiral | 4043 (81.8%) | 229 (85.8%) | 3814 (81.5%) |
| Missing | 1 (0.0%) |  | 1 (0.0%) |
| Number of CT rows |  |  |  |
| <64 | 1365 (27.6%) | 50 (18.7%) | 1315 (28.1%) |
| >=64 | 3581 (72.4%) | 217 (81.3%) | 3364 (71.9%) |
| Experience |  |  |  |
| Neuroradiologist | 1044 (21.1%) | 40 (15.0%) | 1004 (21.5%) |
| Radiology consultant | 2958 (59.8%) | 166 (62.2%) | 2792 (59,7%) |
| Experienced resident | 944 (19.1%) | 61 (22.8%) | 883 (18.9%) |
| CT Indication |  |  |  |
| Neurological symptoms | 2292 (46.3%) | 88 (33.0%) | 2204 (50.5%) |
| Trauma | 2160 (43.7%) | 154 (57.7%) | 2006 (42.9%) |
| Headaches | 217 (4.4%) | 9 (3.4%) | 208 (4.4%) |
| Post-op | 47 (1.0%) | 3 (1.1%) | 44 (0.9%) |
| Unknown cases | 230 (4.7%) | 13 (4.9%) | 217 (4.6%) |
