# Supplementary material for: Deep learning algorithm in detecting intracranial hemorrhages on emergency computed tomographies
Source: PLoS One. 2021 Nov 29;16(11):e0260560. doi: 10.1371/journal.pone.0260560 (PMC8629230; doi:10.1371/journal.pone.0260560)
Supplement: S1 File — (ZIP) [file pone.0260560.s008.zip › Vote Ethics board - translation.pdf]

Ethics committee

Medical Association Berlin (Ärztekammer Berlin)

**Study Title:** *Artifizielle Erkennung intrakranieller Blutungen in notfallmäßigen Computertomographien einer Radiologie und Neuroradiologie mit Teleradiologie*

“Artificial Detection of Intracranial Hemorrhages on Emergency Computertomographies of a Radiology and Neuroradiology Department with Teleradiology”

**Proposal date:** September 24$^{th}$, 2020; Revision Version 1.1 November 25$^{th}$, 2020

**Internal registration number:** Eth-46/20

**Statements of the Ethics committee:**

the ethics board examined your proposal to conduct the above- named study during a meeting on 10/28/2020.

1$^{st}$ Vote (28/10/2020): there are no fundamental concerns about the conduction of the study if the proposed recommendations are addressed.

2$^{nd}$ Vote (12/03/2020): the revised version 1.1 (25.11.2020) implements all recommendations of the first vote from 10/28/2020.
