# Supplementary material for: Deep learning algorithm in detecting intracranial hemorrhages on emergency computed tomographies
Source: PLoS One. 2021 Nov 29;16(11):e0260560. doi: 10.1371/journal.pone.0260560 (PMC8629230; doi:10.1371/journal.pone.0260560)
Supplement: S1 File — (ZIP) [file pone.0260560.s008.zip › First Ethics statement_German.pdf]

ETHIK-KOMMISSION

ÄRZTEKAMMER BERLIN

ÄRZTEKAMMER BERLIN Friedrichstraße 16 · 10969 Berlin

Körperschaft des öffentlichen Rechts

Persönlich/Vertraulich

Herrn Prof. Dr. med. Sven Mutze
Institut für Radiologie und
Neuroradiologie
Unfallkrankenhaus Berlin
Warener Str. 7
12683 Berlin

Ansprechpartner Frau Jasper

Telefon 0 30 / 4 08 06 – 26 46
Zentrale 0 30 / 4 08 06 - 0
Fax 0 30 / 4 08 06 – 22 98

www. aerztekammer-berlin.de

Bitte stets angeben:
Unser Zeichen
Eth-46/20

Berlin, 28.10.2020

Berufsethische und berufsrechtliche Beratung zu Forschungsvorhaben nach § 15 Absatz 1 Berufsordnung

**Titel:**	**Artifizielle Erkennung intrakranieller Blutungen in notfallmäßigen Computertomographien einer Radiologie und Neuroradiologie mit Telera-diologie**
Antragsteller:	Prof. Dr. med. Sven Mutze
Antrag vom:	24.09.2020

Sehr geehrter Herr Prof. Mutze,

der Arbeitsausschuss I der Ethik-Kommission der Ärztekammer Berlin hat Ihren Antrag vom 24.09.2020 auf der Grundlage der von Ihnen vorgelegten Unterlagen geprüft und in den Sitzungen am 28.10.2020 berufsethisch und berufsrechtlich beraten.

V O T U M

Gegen den Beginn des Vorhabens in der vorliegenden Form bestehen

( ) **keine grundlegenden berufsethischen und berufsrechtlichen Bedenken.**

(x) **keine grundlegenden berufsethischen und berufsrechtlichen Bedenken, wenn die in der Anlage enthaltenen Empfehlungen berücksichtigt werden.**

( ) **grundlegende berufsethische / berufsrechtliche Bedenken (siehe ggf. Begründung unter I.)**

I.

Grundlagen der Beratung sind

- die berufsrechtlichen Regelungen
- die in der Deklaration von Helsinki des Weltärztebundes niedergelegten ethischen Grundsätze für die medizinische Forschung am Menschen
- die Empfehlungen der ICH-Guideline for Good Clinical Practice.
