# Supplementary material for: Deep learning algorithm in detecting intracranial hemorrhages on emergency computed tomographies
Source: PLoS One. 2021 Nov 29;16(11):e0260560. doi: 10.1371/journal.pone.0260560 (PMC8629230; doi:10.1371/journal.pone.0260560)
Supplement: S1 File — (ZIP) [file pone.0260560.s008.zip › Final vote_German.pdf]

ETHIK-KOMMISSION

ÄRZTEKAMMER BERLIN

ÄRZTEKAMMER BERLIN Friedrichstraße 16 · 10969 Berlin

Körperschaft des öffentlichen Rechts

Persönlich/Vertraulich

Herrn Prof. Dr. med. Sven Mutze
Institut für Radiologie und
Neuroradiologie
Unfallkrankenhaus Berlin
Warener Str. 7
12683 Berlin

Ansprechpartner Frau Jasper

Telefon 0 30 / 4 08 06 – 26 46
Zentrale 0 30 / 4 08 06 - 0
Fax 0 30 / 4 08 06 – 22 98

www. aerztekammer-berlin.de

Bitte stets angeben:
Unser Zeichen
Eth-46/20

Berlin, 03.12.2020

Berufsethische und berufsrechtliche Beratung zu Forschungsvorhaben nach § 15 Absatz 1 Berufsordnung

**Titel:**	**Artifizielle Erkennung intrakranieller Blutungen in notfallmäßigen Computertomographien einer Radiologie und Neuroradiologie mit Teleradiologie,** hier: Prüfplan Version 1.1 vom 25.11.2020
Antragsteller:	Prof. Dr. med. Sven Mutze

Sehr geehrter Herr Prof. Mutze,

nach Kenntnisnahme des überarbeiteten Prüfplans in der Version 1.1 vom 25.11.2020 bestätigen wir Ihnen, dass damit alle Empfehlungen der Ethik-Kommission aus dem Votum vom 28.10.2020 umgesetzt wurden.

Mit freundlichen Grüßen
Gez.
Dr. med. Johannes Hamann
Vorsitzender des Arbeitsausschusses I
der Ethik-Kommission

Carola Jasper
Abt. 3, Ethik-Kommission

Anlage
