# Supplementary material for: Deep learning algorithm in detecting intracranial hemorrhages on emergency computed tomographies
Source: PLoS One. 2021 Nov 29;16(11):e0260560. doi: 10.1371/journal.pone.0260560 (PMC8629230; doi:10.1371/journal.pone.0260560)
Supplement: S1 File — (ZIP) [file pone.0260560.s008.zip › Study Protocol 4620 AI_Final 25112020_German.pdf]

Studienprotokoll

Artifizielle Erkennung intrakranieller Blutungen in notfallmäßigen Computertomographien einer Radiologie und Neuroradiologie mit Teleradiologie

"Artificial Detection of Intracranial Hemorrhages on Emergency Computertomographies of a Radiology and Neuroradiology Department with Teleradiology"

1. Verantwortlichkeiten

Institutsdirektor:
Prof. Dr. med. Sven Mutze
Unfallkrankenhaus Berlin
Institut für Radiologie und Neuroradiologie

Außerplanmäßiger Professor der
Universitätsmedizin Greifswald

Verantwortliche Ärztin:
Dr. med. Leonie Gölz
Unfallkrankenhaus Berlin
Institut für Radiologie und Neuroradiologie

Promovendin:
Dr. med. Almut Kundisch
Fachärztin für Mund-Kiefer-Gesichtschirurgie

Studienzentrum:
Unfallkrankenhaus Berlin
Institut für Radiologie und Neuroradiologie

2. Wissenschaftlicher Hintergrund

Das BG Klinikum Unfallkrankenhaus Berlin ist ein Krankenhaus der Maximalversorgung mit zertifiziertem, überregionalem Traumazentrum. Dementsprechend wird jährlich eine Vielzahl an Schwerst- und Polytrauma-Verletzten interdisziplinär behandelt. Das Klinikum verfügt über eine neurochirurgische Klinik, die die Akutversorgung leichter bis schwerer Schädel-Hirn-Traumata und Patienten mit intrakraniellen Aneurysmen in Kooperation mit der Neuroradiologie übernimmt. Des weiteren werden auf der überregionalen Stroke-Unit des Klinikums Patienten mit allen anderen Formen intrakranieller Blutungen versorgt.
Das Institut für Radiologie und Neuroradiologie untersucht 24 Stunden am Tag an 7 Tagen in der Woche Patienten nach Schädel-Hirn-Trauma und Patienten mit neurologischer Symptomatik. Zusätzlich zum hohen Patientenaufkommen innerhalb des Klinikums versorgt das Institut für Radiologie und Neuroradiologie 18 weitere

Kliniken im Umland teleradiologisch mit Befunden. Täglich werden daher insgesamt ca. 45-60 cCT‘s notfallmäßig durchgeführt und auf Basis des Facharztstandards befundet.
Aufgrund des hohen Patientenaufkommens zu jeder Tageszeit sind Mechanismen zur Triage und Priorisierung auch im radiologischen Dienstgeschehen notwendig. Nur so kann ein prognostisch entscheidendes, schnelles Handeln im Notfall gewährleistet werden (1). Algorithmen, die standardisierte Untersuchungs- und Behandlungsstrategien ermöglichen sollen, haben sich in vielen Bereichen der Medizin als probate Mittel der Qualitätssicherung etabliert (2). Eine retrospektive Untersuchung zeigte, dass über die teleradiologische Versorgung am häufigsten cCT‘s durchgeführt werden. Insbesondere wird diese Untersuchung an Wochenenden zwischen 8 und 16 Uhr angefordert (3). Struba et al. beschrieben bereits 2007 in einer prospektiven Studie, dass in 1037 von 22.590 Fällen Befunddiskrepanzen zwischen dem Facharzstandard und neuroradiologischen Befunden auftraten. In 0,6% der cCT‘s handelte es sich um Befunddiskrepanzen hinsichtlich intrakranieller Blutungen (4). Häufig kann die neuroradiologische Befundung auch an großen Zentren nicht jederzeit im Nacht- und Wochenenddienst erfolgen und ist auch im Studienzentrum nicht der Standard. Es ist somit im radiologischen Alltag von größter Bedeutung die dringendsten Befunde zuerst zu sichten sowie zu allen Tages- und Nachtzeiten die beste radiologische Versorgung sicherzustellen.
Bereits in den 1940er Jahren wurde in der Literatur die Begriffe „künstliche neuronale Netze“ und „artificial intelligence“ (AI) geprägt (5). Minsky definierte 1968 die „artificial intelligence“ als „the science of making machines do things that would require intelligence if done by men“ (6). Als ein Teilbereich der künstlichen Intelligenz gilt das maschinelle Lernen, in dem Computeralgorithmen anhand von Beispielfällen lernen, bestimmte Aufgaben zu lösen (7). In unzähligen Bereich des alltäglich Lebens unterstützen AI-Prozesse schnelle, komplexe Abläufe und auch in der medizinischen Versorgung spielt künstliche Intelligenz eine zunehmende Rolle. Insbesondere im Fachgebiet der Radiologie wird das Thema künstliche Intelligenz zunehmend als große Chance für Behandler und Patienten verstanden (8). Aufgrund ethischer Bedenken setzt die „Vorbefundung“ durch ein AI-System hierbei nach aktueller Meinung immer die abschließende Beurteilung durch eine Radiologin/ einen Radiologen voraus. In Kliniken mit hohen Untersuchungsvolumina könnten AI-Protokolle jedoch die Sicherheit und Geschwindigkeit der Befundung in Zukunft verbessern.
Eines des modernsten kommerziell erhältlichen und für den europäisch-amerikanischen Markt zugelassenen AI-Systeme wird von der Firma AIDOC (Tel Aviv, Israel) angeboten. Jüngste Studien beschrieben, dass diese Software in der Lage ist intrakranielle Blutungen mit einer Sensitivität von 89 - 95 % und einer Spezifität von 94 - 99 % zu erkennen (9-11,13). In einer bislang noch nicht publizierten Studie von Desbuquoit et al. wurden die Verlässlichkeit der AIDOC-Software und eines Weiterbildungsassistenten im 4. Jahr verglichen. Die Autoren berichten, dass der positive prädiktive Wert der AIDOC Software für die Detektion intrakranieller Blutungen bei 93 % und des Weiterbildungsassistenten bei 99% lag. Sensitivitäten und Spezifitäten wurden jedoch jeweils nicht veröffentlicht (12). Darüber hinaus konnte in einer großen prospektiven Studie durch den Einsatz der AIDOC-Software die Befundungszeit signifikant von 132 ± 193 min auf 73 ± 143 min gesenkt werden (13). Weitere Studienergebnisse deuten darauf hin, dass durch den Einsatz der AIDOC Software 1,6 % mehr intrakranielle Blutungen aufgedeckt werden können (14).

Um die diagnostische Sicherheit zu jeder Tageszeit zu erhöhen und um mit aktuellen und zukünftigen Anforderungen in der Radiologie bestmöglich sowie ressourcensparend umzugehen, wurde die AIDOC-Software zur Detektion intrakranieller Blutungen am Studienzentrum mit Teleradiologie etabliert. In einer groß angelegten, retrospektiven Kohortenstudie sollen nachfolgend die Rate an zusätzlich Erkannten intrakraniellen Blutungen, die Gründe für Fehlbefunde und deren mögliche Bedeutung anhand eines historischen Routine-Datensatzes ermittelt werden.

3. Fragestellung/Hypothese

Primäre Fragestellung:
Wie viele intrakranielle Blutungen werden in der täglichen Klinikroutine durch den Einsatz der AI zusätzlich entdeckt?

Sekundäre Fragestellung:
Wie spezifisch ist die AI-Lösung?

Weitere tertiäre Fragestellungen:
- Was sind die Ursachen für falsche Befunde durch die AI? Größe des Befundes, Lokalisation?
- Wie wirkt sich der Ausbildungsstand auf die Primärbefundung aus?

4. Studientyp

Es handelt sich um eine monozentrische retrospektive Datenauswertung.
Am Studienzentrum wurde eine AI Lösung zur Detektion intrakranieller Blutungen implementiert. Täglich werden durch das Studienzentrum ca. 50 cCTs notfallmäßig befundet.
Retrospektiv sollen Computertomographien des Schädels (cCTs) in einem Zeitraum von vier Monaten vor Implementierung der Software (05/2020-08/2020) eingeschlossen und die Verlässlichkeit der AI evaluiert werden.
Alle Patienten, die in diesem Zeitraum notfallmäßig eine Computertomographie des Schädels (cCT) erhalten haben (ca. n = 5000), werden in Studie eingeschlossen.

5. Zielkriterien

Primär:	Bestimmung divergierender Ergebnisse zwischen der Primärbefundung und den Analysen der AI.
Sekundär:	Bestimmung divergierender Ergebnisse, die durch die AI als negativ erkannt wurden und retrospektive Beurteilung durch den Goldstandard „Neuroradiologin/Neuroradiologe“.
Tertiär:	Bestimmung divergierender Ergebnisse, die durch die AI als positiv erkannt wurden und retrospektive Beurteilung durch den Goldstandard „Neuroradiologin/Neuroradiologe“.

6. Methoden der Datenerhebung (siehe Datenerfassungsbogen)

Recherche in Patientenakten:

- Patientencharakteristika
  - Alter
  - Geschlecht
- Indikation zur Bildgebung:
  - Trauma
  - Vigilanzminderung
  - Kopfschmerz
  - Neurologische Symptomatik
  - unklar
- Therapie der detektierten Blutung im ukb:
  - Neurologische Überwachung/ konservativ
  - Operation
  - Angiographie +/- Embolisation
  - Wechsel von konservativ zu operativ innerhalb von 72 h
  - Infaust
  - keine

- Mortalität durch intrakranielle Blutungen innerhalb des Krankenhausaufenthaltes im ukb

Analyse der Primärbefundung entsprechend Facharztstandard:

- Intrakranielle Blutung vorhanden, ja oder nein?
- Ausbildungsahr des Befunders/der Befunderin

Retrospektive Analyse der historischen Datensätze durch die AI:

- Intrakranielle Blutung vorhanden, ja oder nein?

Vergleich der Primärbefundung mit Resultat der AI:

- Divergenz der Befunde?

Analyse der divergierenden Befunde durch Neuroradiologin/-en:

- Übereinstimmung mit Befundung durch KI oder mit Primärbefund?
  - Verblindung hinsichtlich des Ergebnisses der AI und des Primärbefundes.
  - ➔ Falsche Befunde der AI?
  - ➔ Falsche Primärbefunde?
- Qualität der Bildgebung:
  - Bewegungsartefakte
  - Aufhärtungsartefakte
  - Fremdkörper-Metallartefakte
  - CT-Technik (inkremental, Mehrzeilenspiraltechnik)
- Blutungstyp bei positiven Befunden:
  - subarachnoidal
  - subdural
  - epidural
  - intracerebral

    - intraventrikulär
- Blutungsgröße in größter Ausdehnung:
    - klein < 1mm
    - mittel 1-10 mm
    - offensichtlich > 10 mm
- Blutungslokalisation
    - Supratentoriell
        - Frontal, parietal, okzipital
        - Angrenzend an Kalotte
        - Angrenzend an Schädelbasis
        - Ventrikel
    - Infratentoriell
        - Hirnstamm
        - Kleinhirn
        - Ventrikel
        - Angrenzend an Kalotte
        - Angrenzend an Schädelbasis
- Besonderheiten bzgl. der Schädelkalotte etc.
    - Hyperostosis
    - Dislozierte Kalottenfraktur
    - Verkalkungen
- CT-Technik:
    - Inkremental/Mehrzeilenspiraltechnik
    - Detektortyp/Anzahl der Zeilen

Analyse falscher Befunde der AI (Goldstandard Neuroradiologiebefund):
- Ursachen falscher Befunde: Verkalkungen, Bewegungsartefakte, Aufhärtungsartefakte
- Bewertung des Facharztstandards: Dunkelziffer intrakranieller Blutungen in klinischer Routine, die durch die AI aufgedeckt werden

Studienspezifische Maßnahme/Datenerhebung:
- Studienspezifische Maßnahmen erfolgen nicht. Die Studie evaluiert die AI-Software mit Routine-CT-Daten des Studienzentrums in einem Zeitraum von vier Monaten (24/ 7)

7. Patientencharakteristika

Einschlusskriterien:
1. alle Patienten, die zwischen 05/2020 und 08/2020 notfallmäßig eine native CT des Schädels erhalten haben und die im Institut für Radiologie und Neuroradiologie des Unfallkrankenhaus Berlin befundet wurden
2. Mindestalter 18 Jahre

Ausschlusskriterien:
1. Elektive CT-Untersuchungen des Schädels z.B. Staging-Untersuchungen, Verlaufskontrollen
2. Alter < 18 Jahre

8. Ablauf der Studie

Im Rahmen dieser retrospektiven Erhebung werden zunächst alle Patienten gescreent, die im Zeitraum 05/2020-08/2020 mittels cCT untersucht wurden. Es erfolgt ein Studieneinschluss von 5000 Patienten, die das cCT aufgrund einer Notfallindikation erhalten haben. Minderjährige Patienten werden ausgeschlossen.
Nach Studieneinschluss werden die cCT von einem Algorhythmus basierend auf AI analysiert. Während dieser Analyse arbeitet das Programm verblindet mit Hinblick auf den primären radiologischen Befund. Im Rahmen der Studie erfolgen schon aufgrund des rein retrospektiven Charakters keine zusätzlichen radiologischen Untersuchungen.
Nun wird die Analyse der AI im Rahmen der Studie mit den primären radiologischen. Befunden verglichen. Divergierende Befunde werden Fachärzten der Neuroradiologie zur erneuten Sichtung verblindet vorgelegt. Als Referenzstandard wird durch den Neuroradiologen entschieden, welche Untersuchungen durch die AI falsch oder richtig analysiert wurden.
Durch die AI übersehene Blutungen im Studienkollektiv werden weiter analysiert. Hierbei sind insbesondere Ursachen bzw. Merkmale falscher Analysen interessant. Das Ausmaß der Blutungen und deren Lokalisation sowie Artefakte und die CT-Qualität werden beschrieben. Außerdem wird anhand der Patientenakten am UKB verfolgt, ob primär übersehen Verletzungen im weiteren Behandlungsverlauf auffielen, wie diese behandelt wurden und wie hoch die Mortalität von Hirnblutungen im Patientenkollektiv am UKB war.
Schließlich wird die Anzahl der durch den Einsatz der AI zusätzlich erkannten Hirnblutungen ermittelt.

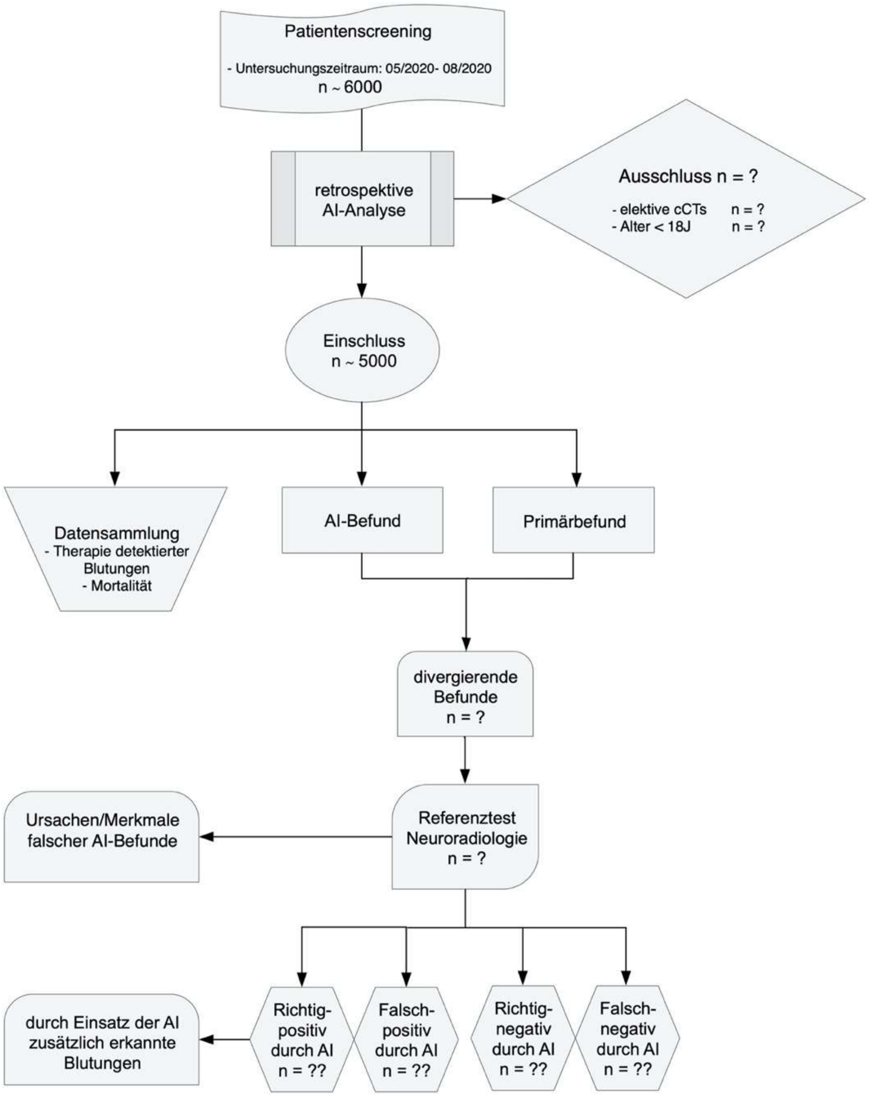

9. Patienten/ Probandensicherheit/ Nebenwirkungen vs. Nutzen

- Das solitäre Risiko der retrospektiven Analyse besteht in der Datenverarbeitung, -speicherung und -veröffentlichung. Maßnahmen zum Datenschutz können dem Punkt 13 entnommen werden.
- Nutzen/ Vorteile: Die Studienergebnisse werden einen Nutzen für die Forschung und die medizinische Gemeinschaft darstellen. Erkenntnisse aus der Studie könnten helfen die radiologische Diagnostik zu jeder Tageszeit und in jedem Institut sicherer zu machen.
- Die Dunkelziffer und Mortalität durch übersehene oder verzögert erkannte intrakranielle Blutungen könnte reduziert werden.
- Der Studieneinschluss bietet keinen individuellen Vorteil für die Patienten.

10. Gesetze/ Verordnungen, die bei dem Vorhaben zu beachten sind

- Berliner Datenschutzgesetz § 17: Verarbeitung personenbezogener Daten zu wissenschaftlichen oder historischen Forschungszwecken und zu statistischen Zwecken
- Berufsordnung der Ärztekammer Berlin § 15 Abs. 1: Pflicht zur Beratung durch eine öffentlich-rechtliche Kommission über die mit der Verarbeitung epidemiologischer Daten verbundenen berufsethischen und berufsrechtlichen Fragen
- Berliner Landeskrankenhausgesetz § 25 Abs. 1 Nr. 1: Die Einholung einer Einwilligungserklärung der Patienten zu diesem Forschungsvorhaben ist entbehrlich, da die Gesundheitsdaten der Patienten im Rahmen der Krankenhausbehandlung innerhalb unserer Fachrichtung erhoben und gespeichert wurden (§ 25 Abs. 1 Nr. 1 LGK Berlin). In diesem Forschungsvorhaben werden lediglich die pseudonymisierten Gesundheitsdaten verwendet. Darüber hinaus stellt die Information an die Patienten über diese Studie einen unverhältnismäßigen Aufwand dar, da die Daten im Rahmen einer Nachbeobachtungsstudie zu wissenschaftlichen Zwecken verwendet werden. Schutzwürdige Belange der Patientin oder des Patienten werden nicht beeinträchtigt (Art. 14 abs. 5 lit. b DSGVO).
- Die geplante Datenerhebung berührt weder das AMG noch das MPG.
    - Alle retrospektiv analysierten CTs wurden im Rahmen der Good Clinical Practice entsprechend des radiologisch Facharztstandards befundet. Studienspezifische Therapien oder Diagnostik fanden nicht statt.

11. Probandenversicherung/ Wegeunfallversicherung

- entfällt für den vorliegenden Studientyp

12. Statistische Planung

Für die geplante retrospektive Auswertung von Routinedaten ist keine formale Fallzahlplanung vorgesehen. Eingeschlossen werden sollen 5000 konsekutive Patienten gemäß den o.g. Ein- und Ausschlusskriterien in einem Zeitraum von vier Monaten (05/2020-08/2020).
Angaben zur Inzidenz aller Formen intrakranieller Blutungen in notfallmäßig durchgeführten cCTs existieren nicht in der Literatur. Bekannt ist aber, dass die Rate an intrakraniellen Blutungen bei Patienten nach mildem Schädel-Hirn-Trauma bei ca. 14% liegt (15). In einem Kollektiv von 5000 Patienten könnten somit bei etwa 700 Personen intrakranielle Blutungen vorliegen. Nimmt man an, dass in ca. 5% der Patienten die Befunde eines allgemeinradiologischen Primärbefunders nicht mit dem Befund eines Neuroradiologen übereinstimmen (16), wären etwa 35 der 5000 Primärbefunde divergent. Laut einer aktuellen Publikation können durch den Einsatz der KI ca. 1,6 % mehr intrakranielle Blutungen erkannt werden (14), bisher noch nicht publizierte Daten des Herstellers deuten sogar auf eine Zunahme der Detektion an intrakraniellen Blutungen um 5-7% hin. Die AI sollte laut diesen Schätzungen somit zwischen 11-49 zusätzliche intrakranielle Blutungen erkennen können.

Basierend auf den o.g. Häufigkeiten erwarten wir eine hinreichend große Datenbasis, um aussagekräftige Schlüsse aus den Auswertungen ziehen zu können.
Die retrospektive Befundung durch eine Neuroradiologin/einen Neuroradiologen wird als Goldstandard zur Detektion intrakranieller Blutungen angesehen. Divergierende Ergebnisse der AI und des Primärbefundes werden mit dem Goldstandard verglichen. Zur korrekten Berechnung der diagnostischen Kennwerte wäre auch eine Überprüfung der konkordanten Befunde von allgemeinradiologischem Primärbefunder und AI Software via Goldstandard nötig, da sonst verzerrte Schätzungen von Sensitivität und/oder Spezifität resultieren können (17). Da aufgrund des zeitlichen Aufwands nicht alle 5000 cCTs erneut durch die Neuroradiologin/den Neuroradiologen gesichtet werden können, ist eine Aussage zur diagnostischen Genauigkeit der AI in Form von Sensitivität, Spezifität, positivem prädiktiven Wert und negativem prädiktivem Wert in diesem Setting somit nicht möglich. Die Aussagen dieser Studie haben keine allgemeine Gültigkeit, sondern werden spezifisch für das untersuchte Patientenkollektiv sein. Es sollen daher lediglich die Häufigkeiten der divergierenden Befunde der AI und der Primärbefundung dargestellt und deren Ursache durch den Goldstandard (Neuroradiologe) erörtert werden.
Die übrigen in den Patientenakten erfassten Parameter werden deskriptiv und in explorativer Manier analysiert. In Abhängigkeit vom Skalenniveau werden absolute und relative Häufigkeiten bzw. Mittelwert, Standardabweichung, Median, 25%- und 75%-Perzentil, Minimum und Maximum berichtet. Alle statistischen Analysen werden mit der SPSS Software V.25 durchgeführt.

13. Datenmanagement/-schutz

- Die Daten werden am Prüfzentrum generiert.
- Alle Daten verbleiben im Studienzentrum. Zugang zu den Daten haben die o.g. Verantwortlichen Ärzte und Biometriker.
- Eine Identifizierung der Patienten ist nur über eine getrennt geführte, papierbasierte Patientenidentifizierungsliste möglich. Nach Abschluss der Datenerhebung vor Beginn der Auswertung erfolgt eine Pseudonymisierung der Daten. Die Daten werden nicht an Dritte weitergegeben
- Studienspezifische Auswertungen werden pseudonymisiert 10 Jahre im Studienzentrum aufbewahrt.
- Die Speicherung des radiologischen Bildmaterials und der Auswertung durch die KI erfolgt im PACS (Picture Archiving and Communication System) des Instituts entsprechend gängiger Standards.
    - Zugangsberechtigt sind nur die Mitarbeiter des Instituts

14. Ethisch-rechtliche Überlegungen

- Die Untersuchung wird in Übereinstimmung mit der Deklaration von Helsinki in ihrer aktuellen Fassung durchgeführt.
- Die Studieninstitution hat sich den Grundsätzen der Guten Wissenschaftlichen Praxis der Deutschen Forschungsgemeinschaft (DFG) verschrieben.
- Die Namen der Patienten und alle anderen vertraulichen Informationen unterliegen der ärztlichen Schweigepflicht und den Bestimmungen der

Datenschutz-Grundverordnung (DSGVO) sowie des Landes- bzw. Bundesdatenschutzgesetzes (LDSG bzw. BDSG).

- Auf das Einholen einer Einverständniserklärung zur Datenverarbeitung muss verzichtet werden, da die Studie retrospektiv erfolgt und eine große Anzahl an Patienten eingeschlossen wird, um die Verlässlichkeit der AI zu prüfen. Bei einer solch hohen Patientenzahl (n ~ 5000) kann nur mit unverhältnismäßig hohem Aufwand die Aufklärung und Einholung einer schriftlichen Einwilligung erfolgen. Wir sehen keinen anderen Weg unseren Forschungszweck zu erreichen.
- Der potenzielle Nutzen dieses Forschungsvorhabens überwiegt das Interesse der betroffenen Patienten an einem Ausschluss der Datenverarbeitung.
- Die auszuwertenden Daten sind im Institut bereits vorhanden, da die ursprüngliche Erhebung im Rahmen der medizinischen Routineversorgung erfolgte. Die Daten sollen lediglich zu Forschungszwecken weiterverarbeitet werden.
- Zum Zweck der Forschung nehmen nur Personen Einsicht in personenbezogene Daten, die bereits zuvor im Rahmen der medizinischen Routineversorgung zur Einsicht berechtigt waren. Biometriker und die Promovendin werten pseudonymisierte Daten aus.
- Eine Weitergabe personenbezogener Daten an externe Stellen erfolgt nicht. Dritte erhalten keinen Einblick in Originalunterlagen.
- Die Datenverarbeitung erfolgt in pseudonymisierter Form. Eine Entschlüsselung erfolgt nur, soweit der Forschungszweck dies erfordert.
- Die Studie wird in einem Register für klinische Studien DRKS vor Beginn der Datenerhebung registriert.
- Die Studie wird in einem peer reviewed Journal unabhängig vom Ergebnis publiziert.
- Der Studieneinschluss bietet keinen individuellen Vorteil für die Patienten. In Fällen falscher Primärbefunde wird jedoch Kontakt mit der Patienten/dem Patienten aufgenommen und ggf. eine ärztliche Vorstellung empfohlen. Diese Maßnahme ist nicht Teil der Studie, sondern ist Ausdruck der ärztlichen Sorgfaltspflicht und wird im ethischen Kontext als notwendig angesehen.

15. Die Projektleiter bestätigen mit der Unterschrift,

- dass auch bei einer positiven Beurteilung des Vorhabens durch die Ethikkommission die ärztliche und juristische Verantwortung uneingeschränkt bei dem/ der Projektleiter/in und seinen Mitarbeiter/innen verbleibt.
- die Richtigkeit und Vollständigkeit dieses Antrages.

Prof. Dr. med. Sven Mutze Dr. med. Leonie Gölz

Anlagen:

- Datenerfassungsbogen CRF_AI Version 1.0

Literatur

1. Kanz KG, Körner M, Linsenmaier U, Kay MV, Huber-Wagner SM, Kreimeier U, Pfeifer KJ, Reiser M, Mutschler W: Prioritätenorientiertes Schockraummanagement unter Integration des Mehrschichtspiralcomputertomographen. Unfallchirurg 2004 · 107:937–944
2. Kanz KG, Eitel F, Waldner H, Schweiberer L (1994) Entwicklung von klinischen Algorithmen für die Qualitätssicherung in der Polytraumaversorgung. Unfallchirurg 97:303–307
3. Schreyer, A.G., Elgharbawy, M., Dendl, L.M. et al. Charakterisierung teleradiologisch untersuchter Patienten an einem Krankenhaus der Schwerpunktversorgung. Radiologe **60,** 729–736 (2020).
4. Struba WM, Leacha JL, Tomsicka T, Vagala A (2007) Overnight preliminary head CT interpretations provided by residents: Locations of misidentified intracranial hemorrhage. Am J Neuroradiol. 28: 1679 - 1682.
5. McCarthy J, Minsky ML, Rochester N, Shannon CE (2006) A proposal for the Dartmouth summer research project on artificial intelligence, August 31,1955.AIMag27(4):12
6. Minsky M (Hrsg) (1968) Semantic information processing.MITPress,Cambridge.
7. Langs G, Attenberger U, Licandro R, Hofmanninger J, Perkonigg M, Zusag M, Röhrich S, Sobotka D, Prosch H: Maschinelles Lernen in der Radiologie. Begriffsbestimmung vomE inzelzeitpunkt bis zur Trajektorie. Radiologe 2020; 60:6–14
8. Beregi JP, Zins M, Masson JP, et al. Radiology and artificial intelligence: An opportunity for our specialty. Diagn Interv Imaging. 2018;99(11):677-678. doi:10.1016/j.diii.2018.11.002
9. Ginat DT. Analysis of head CT scans flagged by deep learning software for acute intracranial hemorrhage. Neuroradiology. 2020;62(3):335-340. doi:10.1007/s00234-019-02330-w.
10. P. Ojeda, M. Zawaideh, M. Mossa-Basha, D. Haynor, "The utility of deep learning: evaluation of a convolutional neural network for detection of intracranial bleeds on non-contrast head computed tomography studies," Proc. SPIE 10949, Medical Imaging 2019: Image Processing, 109493J (15 March 2019).
11. Raskin E, Yaniv G , Hoffmann C, Konen E. Preliminary Results of AIDOC's Deep Learning Algorithm Detection Accuracy for Pathological Intracranial Hyperdense Lesions. Kongressbeitrag Israel Radiological Association Annual Meeting 2018. https://program.eventact.com/lecture?id=183035&code=2504404.
12. Desbuquoit D, Dekeyzer S, Huyskens J, Nicolay S, De Smet E, Van Goethem J, Van den Hauwe L, Parizel PM. Detection of Intracranial Haemorrhage on CT of the Brain Using A Deep Learning Algorithm. Edegem/BE. https://www.aidoc.com/blog/clinical_study/detection-of-intracranial-haemorrhage-on-ct-of-the-brain-using-a-deep-learning-algorithm.
13. Axel Wismüller, Larry Stockmaster, "A prospective randomized clinical trial for measuring radiology study reporting time on Artificial Intelligence-based detection of intracranial hemorrhage in emergent care head CT," Proc. SPIE 11317, Medical Imaging 2020: Biomedical Applications in Molecular, Structural, and Functional Imaging, 113170M (28 February 2020);https://doi.org/10.1117/12.2552400.
14. Rao B, Zohrabian V, Cedeno P, Saha A, Pahade J, Davis MA. Utility of Artificial Intelligence Tool as a Prospective Radiology Peer Reviewer - Detection of Unreported Intracranial Hemorrhage [published online ahead of print, 2020 Feb 24]. Acad Radiol. 2020;S1076-6332(20)30084-2. doi:10.1016/j.acra.2020.01.035
15. Yuksen C, Sittichanbuncha Y, Patumanond J, Muengtaweepongsa S, Sawanyawisuth K. Clinical predictive score of intracranial hemorrhage in mild traumatic brain injury. Ther Clin Risk Manag. 2018;14:213-218. Published 2018 Feb 1. doi:10.2147/TCRM.S147079
16. William K, Boyd C. Ashdown, Richard W. Lucio, II, Raymond F. Carmody, Joachim F. Seeger, and Jennifer N. Alcala. American Journal of Roentgenology 2003 180:6, 1727-1730.
17. U.S. Food & Drug Administration. Center for Devices and Radiological Health. Statistical Guidance on Reporting Results from Studies Evaluating Diagnostic Tests - Guidance for Industry and FDA Staff. FDA-2020-D-0957. March 2007. https://www.fda.gov/regulatory-information/search-fda-guidance-documents/statistical-guidance-reporting-results-studies-evaluating-diagnostic-tests-guidance-industry-and-fda.
