# Supplementary material for: Deep learning algorithm in detecting intracranial hemorrhages on emergency computed tomographies
Source: PLoS One. 2021 Nov 29;16(11):e0260560. doi: 10.1371/journal.pone.0260560 (PMC8629230; doi:10.1371/journal.pone.0260560)
Supplement: S1 File — (ZIP) [file pone.0260560.s008.zip › CRF - Translation.pdf]

Institut für Radiologie und Neuroradiologie

Prof. Dr. Sven Mutze
Institutsdirektor
Telefon: +49 30 56 81-3801
Telefax: +49 30 56 81-3803

Unfallkrankenhaus Berlin

Case Report Form

"Artificial Detection of Intracranial Hemorrhages on Emergency Computed Tomographies of a Radiology and Neuroradiology Department with Teleradiology"

Subject-ID	
Location of CT examination	☐ In house<br>☐ Teleradiology
Age	
CT-Technique	☐ Incremental<br>☐ Spiral
Detector, number of rows	
gender	☐ female<br>☐ male
Indication for CT	☐ trauma<br>☐ reduced vigilance<br>☐ headaches<br>☐ neurological symptoms<br>☐ uncertain
Therapy of ICH	☐ surveillance/conservative<br>☐ operation<br>☐ Angiography +/- Embolization<br>☐ switch conservative to operation<br>☐ infaust<br>☐ uncertain<br>☐ none
Death	☐ Yes<br>☐ No
Primary radiology report/CH?	☐ Yes<br>☐ No<br>☐ Uncertain
Experience of primary radiologist in years	
Result of AI/ICH?	☐ Yes<br>☐ No
Gold Standard (neuroradiologist)/ICH?	☐ Yes<br>☐ No
Congruent result of AI an neuroradiologist?	☐ Yes<br>☐ No

Additional ICH detected by AI?	☐ Yes<br>☐ No
Artifacts?	☐ motion<br>☐ beam-hardening<br>☐ metal
Type of ICH	☐ subarachnoid<br>☐ subdural<br>☐ epidural<br>☐ intracerebral<br>☐ intraventricular
Max. size of ICH	☐ small < 1mm<br>☐ medium 1-10 mm<br>☐ obvious > 10 mm
Localization of ICH	☐ Supratentorial<br>○ Frontal<br>○ Parietal<br>○ Occipital<br>○ Ventricle<br>☐ Infratentorial<br>○ Brain stem<br>○ Cerebellum<br>○ Ventricle<br>☐ Adjacent to<br>○ vertex<br>○ scull base<br>○ Ventricle
Extra-ordinary FIndings	☐ Hyperostosis<br>☐ dislocated fracture<br>☐ calcifications<br>○ Ventricle<br>○ Basal ganglia<br>○ Parenchyma supratent.<br>○ Parenchyma infratent.
Patient contacted?	☐ Yes<br>☐ No
