# Supplementary material for: Deep learning algorithm in detecting intracranial hemorrhages on emergency computed tomographies
Source: PLoS One. 2021 Nov 29;16(11):e0260560. doi: 10.1371/journal.pone.0260560 (PMC8629230; doi:10.1371/journal.pone.0260560)
Supplement: S1 File — (ZIP) [file pone.0260560.s008.zip › CRF_V 1.0_German.pdf]

Institut für Radiologie und Neuroradiologie

Prof. Dr. Sven Mutze
Institutsdirektor
Telefon: +49 30 56 81-3801
Telefax: +49 30 56 81-3803

Unfallkrankenhaus Berlin

Datenerfassung

Verlässlichkeit der artifiziellen Erkennung intrakranieller Blutungen in notfallmäßigen Computertomographien einer Radiologie und Neuroradiologie mit Teleradiologie

Subject-ID	
Ort der Bildgebung	☐ In house<br>☐ Teleradiologie
Alter	
CT-Technik	☐ Inkremental<br>☐ Mehrzeilenspiraltechnik
Detektortyp/Anzahl der Zeilen	
Geschlecht	☐ Weiblich<br>☐ Männlich
cCT-Indikation	☐ Trauma<br>☐ Vigilanzminderung<br>☐ Kopfschmerz<br>☐ Neurologische Symptomatik<br>☐ unklar
Therapie der detektierten Blutung	☐ Neurologische Überwachung/konservativ<br>☐ Operation<br>☐ Angiographie +/- Embolisation<br>☐ Wechsel von konservativ zu operativ<br>☐ Infaust<br>☐ unklar<br>☐ keine
Verstorben	☐ Ja<br>☐ Nein
Primärbefund des Radiologen/der Radiologin im Dienst entsprechend Facharztstandard (Blutung?)	☐ Ja<br>☐ Nein<br>☐ Unsicher
Ausbildungsjahr Primärbefunder/-befunderin	
Befund der AI/Blutung?	☐ Ja<br>☐ Nein
Befund Goldstandard (Neuroradiologin/-e)/Blutung?	☐ Ja<br>☐ Nein
Übereinstimmung Neuroradiologin/-e mit AI-Befund?	☐ Ja<br>☐ Nein

Aufdeckung falsch-negativer Primärbefunde durch die KI erfolgt?	☐ Ja<br>☐ Nein
Artefakte?	☐ Bewegungsartefakte<br>☐ Aufhärtungsartefakte<br>☐ Fremdkörper-Metallartefakte
Blutungstyp	☐ subarachnoidal<br>☐ subdural<br>☐ epidural<br>☐ intracerebral<br>☐ intraventrikulär
Blutungsgröße in größter Ausdehnung	☐ klein < 1mm<br>☐ mittel 1-10 mm<br>☐ offensichtlich > 10 mm
Blutungslokalisation	☐ Supratentoriell<br>○ Frontal<br>○ Parietal<br>○ Okzipital<br>○ Ventrikel<br>☐ Infratentoriell<br>○ Hirnstamm<br>○ Kleinhirn<br>○ Ventrikel<br>☐ Angrenzend an<br>○ Kalotte<br>○ Schädelbasis<br>○ Ventrikel
Besonderheiten	☐ Hyperostosis<br>☐ Dislozierte Kalottenfraktur<br>☐ Verkalkungen<br>○ Ventrikel<br>○ Stammganglien<br>○ Parenchym supratent.<br>○ Parenchym infratent.
Kontakt zum Patient aufgrund Studienergebnis notwendig?	☐ Ja<br>☐ Nein
