# Supplementary material for: Deep learning algorithm in detecting intracranial hemorrhages on emergency computed tomographies
Source: PLoS One. 2021 Nov 29;16(11):e0260560. doi: 10.1371/journal.pone.0260560 (PMC8629230; doi:10.1371/journal.pone.0260560)
Supplement: S1 File — (ZIP) [file pone.0260560.s008.zip › Study protocol summary - translation.pdf]

Study Protocol

"Artificial Detection of Intracranial Hemorrhages on Emergency Computed Tomographies of a Radiology and Neuroradiology Department with Teleradiology"

1. Responsible Personnel

Director:
Prof. Dr. med. Sven Mutze
Unfallkrankenhaus Berlin
Institut für Radiologie und Neuroradiologie

Conducting doctor:
Dr. med. Leonie Gölz
Unfallkrankenhaus Berlin
Institut für Radiologie und Neuroradiologie

Ph.D. Student:
Dr. med. Almut Kundisch
Fachärztin für Mund-Kiefer-Gesichtschirurgie

Study center:
Unfallkrankenhaus Berlin
Institut für Radiologie und Neuroradiologie

2. Background (– as relevant for protocol)

To increase diagnostic accuracy during any time of the day and to increase efficiency of our radiology department, the AIDOC AI solution for detection of ICH was implemented at the study site with large teleradiology. In a retrospective cohort-study we aim to analyze the number of additionally detected ICH by the AI and to find reasons for false results and their possible consequences.

3. Hypothesis/Questions

Primary Hypothesis:
How many additional ICH can be detected in daily routine by the AI?

Secondary Hypothesis:
How specific is the AI solution?

Additional tertiary questions:
- What are reasons for false results of the AI (size, location)?
- Does experience of radiologists influence primary reports?

4. Study Type

Retrospective study conducted from one study site but including imaging from all 17 teleradiologies. Screening of cases before implementation of the AI solution to include app. 5000 cases (05/2020-08/2020) to evaluate the reliability of the AI.

5. Aims

Primary: Identify the discrepant results of primary report and AI.
Secondary: Comparison of negative AI results with neuroradiological re-evaluation
Tertiary: Comparison of positive AI results with neuroradiological re-evaluation

6. Methods

Patient records (*see CRF for data acquisition)*

Analysis of primary report:
- ICH?
- Experience of radiologist

Retrospective analysis by AI:
- ICH?

Comparison of results of primary report and AI:
- Discrepant results?

Analysis of discrepant results by neuroradiologist:
- ICH?
  - Blinded to results of AI and radiology report d

  ➔ False results of AI?

  ➔ False primary results?
- Quality of CT:
  - Motion artifacts
  - Beam hardening artifacts
  - Metal artifacts
  - CT- technique (incremental, spiral), number of rows
- Type of ICH for positive cases:
  - subarachnoid
  - subdural
  - epidural
  - intracerebral
  - intraventricular
- max size of ICH:
  - < 1mm
  - 1-10 mm
  - > 10 mm
- Localization of ICH
  - Supratentorial

        - Frontal, parietal, occipital
        - Adjacent to vertex, scull base, ventricle
    - Infratentorial
        - Brain stem
        - cerebellum
        - Ventricle
        - Adjacent to scull base, ventricle
- Specifics
    - Hyperostosis
    - Dislocated fracture
    - calcifications

Analysis of false results (gold standard neuroradiologist):
- Reasons for false results: calcifications motion artifacts, beam hardening
- Underreporting in clinical routine, additionally detected ICH by AI

Study specific issues:
- None. All patients received the diagnostic and therapeutic standard of care.

7. Patients

Inclusion:
1. patients who received an emergent head CT (05/2020 - 08/2020) and report from the study site
2. minimum 18 years of age

Exclusion:
1. Non-emergent head CT
2. minors

8. Study Process

Screening of all patients with head CT at study site and teleradiologies between 05/2020-08/2020. Inclusion of emergency cases, exclusion of minors.
Analysis of cases by the AI. The AI will not be provided with primary reports or indications. Comparison of AI results and primary radiology report. Discrepant results will be re-evaluated by neuroradiologists in a blinded fashion as reference test (gold standard). Analysis of false results: size of ICH, localization, artifacts, imaging quality. Consultation of patient records for therapy of patients and mortality at the study site. Finally, calculation of additionally detected ICH by the AI algorithm.

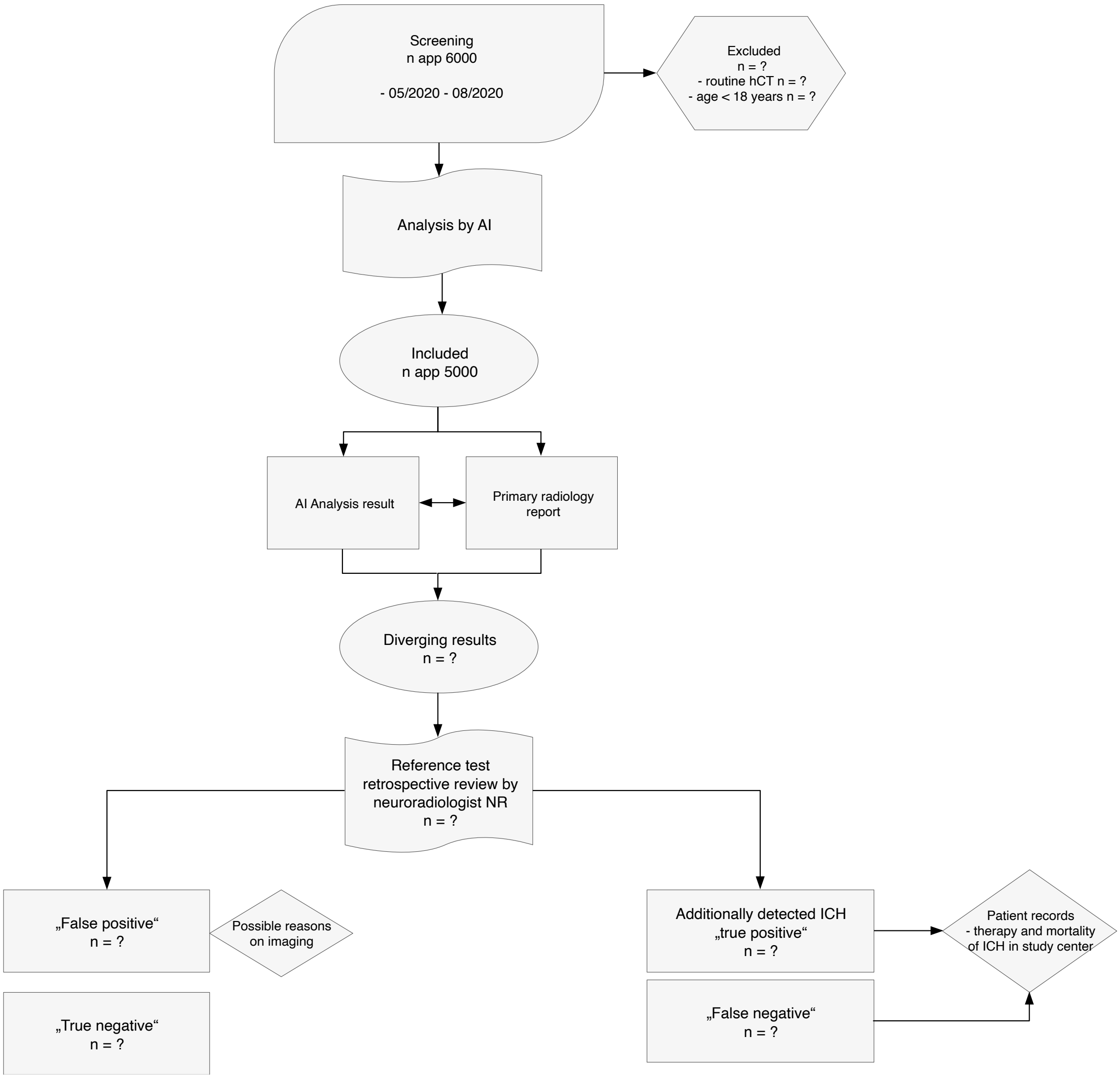

9. Safety/Side effects/benefits

- risks only concern data security (see 13.)
- benefits: improvement of radiological care through insights into AI solutions, reduction of underreporting through implementation of AI at other centers
- no individual benefits for included patients

10. Applicable Law

- Berliner Datenschutzgesetz § 17: data handling for scientific reasons
- Berufsordnung der Ärztekammer Berlin § 15 Abs. 1: Duty to consult an ethics commission
- Berliner Landeskrankenhausgesetz § 25 Abs. 1 Nr. 1
- The study doesn’t include testing of drugs or unapproved devices
- Good Clinical Practice, standard of care

11. Insurance

- Not necessary

12. Statistical planning

Retrospective analysis without pre-calculation of number of cases. Inclusion of app. 5000 consecutive cases (05/2020-08/2020).
Rate of ICH after mild trauma is app. 14% (15). In a collective with 5000 patients, 700 ICH could be found. App. 5% of primary reports do not match re-evaluation by neuroradiologists (16), so 35 of 5000 results could be discrepant. A study reported that the AI solution can detect 1,6 % additional ICH (14), unpublicized data suggests a rate of up to 5-7%. The AI algorithm could detect 11-49 additional ICH. We conclude that 5000 cases should suffice to answer our questions.
We are aware that to calculate true diagnostic values re-evaluation of all cases by a neuroradiologists would be necessary (17) but are too time-consuming in this study setting. The results of the study will be specific for our case mix and will evaluate the number of discrepant cases and the reasons for discrepancies.
Parameters from patient records will be described in an explorative fashion resulting in absolute and relative values, mean, standard deviation, median, confidence intervals, minimum, and maximum. Statistical analysis will be conducted using SPSS Software V.25.

13. Data security

- Data will be generated at the (main) study site (ukb), remain there, and will not be passed to third parties
- Data can only be accessed by responsible/involved doctors and statistician
- Identification of cases is possible only through a separated list for de-pseudonymization
- Imaging will be stored in the PACS (Picture Archiving and Communication System) of the study sites

14. Ethics

- The study will be conducted in accordance with the declaration of Helsinki in it’s most recent form
- The study center adheres to the principles of Good Clinical Practice of the German Scientific Society (DFG)
- All data is protected by professional secrecy and privacy protection laws of state and country (DSGVO, LDSG, BDSG).
- Written consent cannot be obtained due to the retrospective nature of the study and the necessity for a large design (app. 5000 patients) to test the reliability of the AI
- The potential scientific benefit of the study will outweigh the risks of managing existing data
- Involved personal was involved in patient care, Ph.D. student and statistician will handle pseudonymized data

- Data will not be transferred to third parties. Original data cannot be accessed by third parties.
- Data will be handled in pseudonymized forms whenever possible
- The study will be registered prospectively at a clinical trials registry (DRKS)
- The study will be publicized in a peer reviewed journal independent of its results
- In cases of additional ICH, patients will be advised to seek follow-up imaging/care, if necessary, as part of routine medical care but not of as part this study

15. Signature

- The responsibility for the study remains with the conducting personnel
- This application is complete and accurate

Prof. Dr. med. Sven Mutze Dr. med. Leonie Gölz

Attachement:

- CRF_AI Version 1.0

Literature

1. Kanz KG, Körner M, Linsenmaier U, Kay MV, Huber-Wagner SM, Kreimeier U, Pfeifer KJ, Reiser M, Mutschler W: Prioritätenorientiertes Schockraummanagement unter Integration des Mehrschichtspiralcomputertomographen. Unfallchirurg 2004 · 107:937–944
2. Kanz KG, Eitel F, Waldner H, Schweiberer L (1994) Entwicklung von klinischen Algorithmen für die Qualitätssicherung in der Polytraumaversorgung. Unfallchirurg 97:303–307
3. Schreyer, A.G., Elgharbawy, M., Dendl, L.M. et al. Charakterisierung teleradiologisch untersuchter Patienten an einem Krankenhaus der Schwerpunktversorgung. Radiologe **60,** 729–736 (2020).
4. Struba WM, Leacha JL, Tomsicka T, Vagala A (2007) Overnight preliminary head CT interpretations provided by residents: Locations of misidentified intracranial hemorrhage. Am J Neuroradiol. 28: 1679 - 1682.
5. McCarthy J, Minsky ML, Rochester N, Shannon CE (2006) A proposal for the Dartmouth summer research project on artificial intelligence, August 31,1955.AIMag27(4):12
6. Minsky M (Hrsg) (1968) Semantic information processing.MITPress,Cambridge.
7. Langs G, Attenberger U, Licandro R, Hofmanninger J, Perkonigg M, Zusag M, Röhrich S, Sobotka D, Prosch H: Maschinelles Lernen in der Radiologie. Begriffsbestimmung vomE inzelzeitpunkt bis zur Trajektorie. Radiologe 2020; 60:6–14
8. Beregi JP, Zins M, Masson JP, et al. Radiology and artificial intelligence: An opportunity for our specialty. Diagn Interv Imaging. 2018;99(11):677-678. doi:10.1016/j.diii.2018.11.002
9. Ginat DT. Analysis of head CT scans flagged by deep learning software for acute intracranial hemorrhage. Neuroradiology. 2020;62(3):335-340. doi:10.1007/s00234-019-02330-w.
10. P. Ojeda, M. Zawaideh, M. Mossa-Basha, D. Haynor, "The utility of deep learning: evaluation of a convolutional neural network for detection of intracranial bleeds on non-contrast head computed tomography studies," Proc. SPIE 10949, Medical Imaging 2019: Image Processing, 109493J (15 March 2019).

11. Raskin E, Yaniv G , Hoffmann C, Konen E. Preliminary Results of AIDOC's Deep Learning Algorithm Detection Accuracy for Pathological Intracranial Hyperdense Lesions. Kongressbeitrag Israel Radiological Association Annual Meeting 2018. https://program.eventact.com/lecture?id=183035&code=2504404.
12. Desbuquoit D, Dekeyzer S, Huyskens J, Nicolay S, De Smet E, Van Goethem J, Van den Hauwe L, Parizel PM. Detection of Intracranial Haemorrhage on CT of the Brain Using A Deep Learning Algorithm. Edegem/BE. https://www.aidoc.com/blog/clinical_study/detection-of-intracranial-haemorrhage-on-ct-of-the-brain-using-a-deep-learning-algorithm.
13. Axel Wismüller, Larry Stockmaster, "A prospective randomized clinical trial for measuring radiology study reporting time on Artificial Intelligence-based detection of intracranial hemorrhage in emergent care head CT," Proc. SPIE 11317, Medical Imaging 2020: Biomedical Applications in Molecular, Structural, and Functional Imaging, 113170M (28 February 2020);https://doi.org/10.1117/12.2552400.
14. Rao B, Zohrabian V, Cedeno P, Saha A, Pahade J, Davis MA. Utility of Artificial Intelligence Tool as a Prospective Radiology Peer Reviewer - Detection of Unreported Intracranial Hemorrhage [published online ahead of print, 2020 Feb 24]. Acad Radiol. 2020;S1076-6332(20)30084-2. doi:10.1016/j.acra.2020.01.035
15. Yuksen C, Sittichanbuncha Y, Patumanond J, Muengtaweepongsa S, Sawanyawisuth K. Clinical predictive score of intracranial hemorrhage in mild traumatic brain injury. Ther Clin Risk Manag. 2018;14:213-218. Published 2018 Feb 1. doi:10.2147/TCRM.S147079
16. William K, Boyd C. Ashdown, Richard W. Lucio, II, Raymond F. Carmody, Joachim F. Seeger, and Jennifer N. Alcala. American Journal of Roentgenology 2003 180:6, 1727-1730.
17. U.S. Food & Drug Administration. Center for Devices and Radiological Health. Statistical Guidance on Reporting Results from Studies Evaluating Diagnostic Tests - Guidance for Industry and FDA Staff. FDA-2020-D-0957. March 2007. https://www.fda.gov/regulatory-information/search-fda-guidance-documents/statistical-guidance-reporting-results-studies-evaluating-diagnostic-tests-guidance-industry-and-fda.
