# Supplementary material for: Deep learning algorithm in detecting intracranial hemorrhages on emergency computed tomographies
Source: PLoS One. 2021 Nov 29;16(11):e0260560. doi: 10.1371/journal.pone.0260560 (PMC8629230; doi:10.1371/journal.pone.0260560)
Supplement: Attachment — Submitted filename: Response to Reviewers.pdf [file pone.0260560.s009.pdf]

Dear Editors and Reviewers,

First, we like to thank you for your time and efforts spent on our behalf. Please find attached our revised manuscript as well as a point-by-point response to the reviewers' requests and remarks. To further improve the language of the manuscript, it has now undergone additional professional editing.
We used the "track changes" option to highlight our changes and a "changes made in the manuscript" section in our response letter.

We hope we addressed all issues of concern to your full satisfaction, and that the current manuscript is suitable for another round of peer review and possible publication in the Journal.

Kind regards,

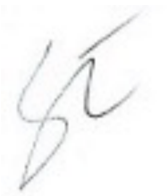

Leonie Goelz

Reviewer	Comment	Response	Changes Made in the Manuscript
1	The authors analyze an interesting topic and the article is written in good English.	Thank you very much for this evaluation! In order to further improve the language to a native speaker level, the manuscript has now undergone additional professional editing.	Throughout.
	The use of artificial intelligence in detecting brain haemorrhages in emergency departments could reduce stress for healthcare workers and residences. It should also reduce the overcall diagnosis rate as well as the Numbers of inappropriate neurosurgical consultation. Therefore the author considered only brain haemorrhages which are only part of the injury in this context as in trauma and in case of loss of consciousness. Although highly skilled workers are needed to maintain the system. The author has not analyzed this topic.	Thank you for raising this interesting topic! To stress the interdisciplinary treatment of many patients by highly specialized personnel and to describe AI analysis of ICH as only one small supportive tool for these teams, we used the example of polytraumatized patients and inserted another paragraph and reference into the discussion.	Page 15, lines 2707-2713.
	The RRs were reports from different centers and provided by radiologists with jeopardized experience and skills . For this reason the comparison does not reflect reals superiority in term of detction rate of AI on resisence and/or radiologists.	Thank you for this comment! AI should only be a supportive tool for radiologists. The results of our study show, that AI analysis is not superior but can be a helpful addition to the clinical routine especially during stressful on-call duty. We added another remark in the discussion to underline our perspective of AI use for ICH detection.<br><br>The comparability of AI and RR performance is represented in Table 1 (page 9). We refrained from statistically comparing the performances or calculating diagnostic accuracy measures because only discrepant results were reevaluated. We elaborated on this limitation in the discussion (page 16, lines 2888-2890).	Page 15, lines 2703-2705.

		We clarified that all primary reports had been composed by personnel trained at the main study site by radiologists and neuroradiologist.	Page 5, lines 805-807.
	Selection Bias in setting of retrospective study should be also considered.	Indeed, it is! We mentioned the possibility of selection bias (discussion, p15 lines 2678-2680) and the measures we took to reduce the probability.	N/A
2	The language needs language polishing throughout the paper.	Thank you for this valuable criticism! The manuscript has now undergone professional editing and revision to address this issue.	Throughout.
	The first sentence in the abstract does not make sense! Rephrase.<br>In general the abstract sounds 'robotic'. Please rewrite in a more vivid way.	Thank you for this suggestion. The abstract was revised by the authors and a professional editor.	Page 2.
	Included cases were analyzed by the AI algorithm.: This is not sufficient for a scientific paper. What algorithm? Why this one? Are there no alternatives? How selected? Details about the analysis.<br><br>The main part of the paper is missing: What is the AI algorithm?	Thank you very much for raising these methodological issues! We address your comments in the methods and discussion sections. Nevertheless, it has to be kept in mind, that details about the underlying programming and training of a commercially available software remain somewhat inscrutable.<br>This particular AI algorithm was chosen among 11 (https://www.statnews.com/wp-content/uploads/2021/02/STAT_FDA_cleared-_AI_tools.xlsx) different, commercially available modules for ICH detection. As consumers, we feel that choosing a product is often challenging and can be overwhelming at times. We became aware of this product at a radiology conference. After researching and reading about the high accuracy of the tool in recent literature, we decided to acquire our first AI solution. The results of this study were meant to identify the benefit of this particular tool for our institute and similarly large centers or teleradiologies retrospectively. We feel that reviewing the	Page 5, lines 816-832.<br><br>Page 16, lines 2886-2890.

		performance of various new AI algorithms could further assist other colleagues in the decision-making process before the implementation of a new AI algorithm but comparative studies between competing products are certainly warranted.	
	Line 159: What is RR analysis?	In the revised manuscript "RR" is now defined in the abstract and main text. Thank you for this remark,	Pages 2/3, lines 190 and 388
3	The aim of this study is to clarify the utility of deep learning algorithm, which is a non-supervised form of artificial intelligence (AI), in detecting intracranial hemorrhages (ICH).<br><br>The article is well written in a correct English.<br><br>As teleradiology networks continue to develop, AI could be a useful tool to screen the images assuring a prompt treatment in emergent cases.<br><br>The study found that AI could increase the number of ICH found in a level 1 trauma center with teleradiology. More interestingly the authors state that in more than 50% of the ICH missed by the radiology, initial diagnosis would have influenced the therapeutic regime.<br>The topic is interesting. AI could improve diagnostic accuracy in the nearest future.	Thank you very much for this very positive evaluation of our work! We appreciate this detailed and pointed summary of the results of this study.	
